# Electrophysiological Studies Revealed CaM1-Mediated Regulation of the *Arabidopsis* Calcium Channel CNGC12

**DOI:** 10.3389/fpls.2019.01090

**Published:** 2019-09-10

**Authors:** Zhengli Zhang, Congcong Hou, Wang Tian, Legong Li, Huifen Zhu

**Affiliations:** College of Life Sciences, Capital Normal University, Beijing, China

**Keywords:** CNGC12, Ca^2+^ channel activity, CaM1, cyclic nucleotide monophosphates, *Arabidopsis*

## Abstract

The *Arabidopsis* cyclic nucleotide-gated channel (CNGC) family consists of 20 members, which have been reported to participate in various physiological processes, such as pathogen defense, development, and thermotolerance. Although CNGC11 and CNGC12 have been identified a decade ago and their role in programmed cell death is well studied, their precise channel regulation has not been studied electrophysiologically. Here, we determined the channel activities of CNGC11 and CNGC12 utilizing the two-electrode voltage-clamp technique in the *Xenopus laevis* oocyte heterologous expression system. Our results suggest that CNGC12 but not CNGC11 functions as an active calcium channel. Furthermore, the cyclic nucleotide monophosphates (cNMPs) did not affect the activities of CNGC11 nor CNGC12 in *Xenopus* oocytes. Interestingly, while the activity of CNGC11 was not affected by co-expression with calmodulin (CaM), the activity of CNGC12 was significantly enhanced when CaM1 was co-expressed in oocytes. This study reveals that the channel activities and the mechanisms of regulation by CaM are different between CNGC11 and CNGC12

## Introduction

Cyclic nucleotide-gated channels (CNGCs) are cationic-permeable ion channels. CNGC channels contain a cytosolic N-terminus, six membrane-spanning domains (S1–S6), a pore domain, and a cytoplasmic C-terminus that includes a cyclic nucleotide-binding domain (CNBD) and a calmodulin-binding domain (CaMBD) (Zagotta and Siegelbaum, 1996; Kaupp and Seifert, 2002; Talke et al., 2003; Yoshioka et al., 2006). CNGC channels, first discovered in animals, crucially function in light and scent signal transduction (Kaupp and Seifert, 2002). There are only six CNGC isoforms reported in mammals (Kaupp and Seifert, 2002), but many more reported in plant species.

In *Arabidopsis thaliana*, there are 20 members in the CNGC family, which are thought to participate in various physiological processes, such as biotic and abiotic stresses and plant development and growth (Clough et al., 2000; Chan et al., 2003; Dietrich et al., 2010; Ma and Berkowitz, 2011). For example, CNGC5 and CNGC6 were identified as Ca^2+^-permeable channels in guard cells (Wang et al., 2013). CNGC2 is located on the plasma membrane (PM) and functions as a calcium-permeable channel that plays key roles in defense response and senescence signaling (Ma et al., 2010). CNGC2 and CNGC4 are implicated in pathogen defense and floral transition (Clough et al., 2000; Chin et al., 2013). CNGC18 is involved in pollen germination and pollen tube growth and has Ca^2+^-permeable cation channel activity in *Arabidopsis* (Frietsch et al., 2007; Zhou et al., 2014; Gu et al., 2017; Pan et al., 2019). CNGC14 is involved in mediating calcium influx during tip growth in root hairs (Zhang et al., 2017). CNGC6, CNGC9, and CNGC14 were reported to maintain cytosolic Ca^2+^ oscillations and polar growth of root hairs (Brost et al., 2019). CNGC10 mediates Ca^2+^ and Mg^2+^ transports in *Arabidopsis* (Guo et al., 2010). CNGC11 and CNGC12 are known to be involved in plant immunity and physiological responses in a Ca^2+^-dependent manner (Yoshioka et al., 2006; Urquhart et al., 2007; Chin et al., 2010; Urquhart et al., 2011; Moeder et al., 2019). Previous studies have shown that both CNGC11 and CNGC12 participated in Ca^2+^ transport using a yeast heterologous expression system (Urquhart et al., 2007; Chin et al., 2010). Some CNGCs contribute to heavy metal ion (Cd^2+^ and Pb^2+^) uptake (Moon et al., 2019). Animal CNGCs are non-selective cation channels which are gated by the second massager cyclic nucleotide monophosphates [cNMPs; 3′,5′-cyclic AMP (3′,5′-cAMP) and 3′,5′-cyclic guanosine monophosphate (3′,5′-cGMP)] (Brüggemann et al., 1993; Kaupp and Seifert, 2002), and some *Arabidopsis* CNGCs have been reported to function as a cyclic nucleotide-gated Ca^2+^-permeable channel (Leng et al., 2002; Ali et al., 2007; Gao et al., 2012; Wang et al., 2013). Previous studies have shown that CNGC11 and CNGC12 are activated by cAMP but not by cGMP (Yoshioka et al., 2006). However, direct evidence supporting this is currently lacking *in planta*. Earlier reports have hypothesized that plant CNGCs possess a single CaMBD, which overlaps with the CNBD in the C-terminus (Kaupp and Seifert, 2002). While recent studies showed that CNGC12 has multiple CaMBDs at the cytosolic N- and C-termini (DeFalco et al., 2016), much like animal CNGCs but unlike any plant CNGC channels studied to date (DeFalco et al., 2016). A motif rich in conserved isoleucine–glutamine, the IQ motif, was first identified as IQxxxRGxxxR (Rhoads and Friedberg, 1997) and has been updated to [MFILV]QXXXRXXXX[RK] (Bähler and Rhoads, 2002), which is conserved in most plant CNGCs. Recent studies show that CaM can bind to the IQ motif of CNGC20 (Fischer et al., 2013) in both the apo- and Ca^2+^-loaded CaMs and can both positively and negatively regulate CNGC12 (DeFalco et al., 2016). Although different isoforms of CNGC12 were assayed by different methods under different conditions, most have not been amenable to direct electrophysiological analysis (DeFalco et al., 2016).

In this study, using two-electrode voltage clamp (TEVC) technology, we demonstrate that CNGC12, but not CNGC11, is an active Ca^2+^-permeable channel in *Xenopus* oocytes. Furthermore, the activity of CNGC11 or CNGC12 is not affected by cNMPs. CaM1 interacts with and activates CNGC12.

## Materials and Methods

### Plant Materials and Growth Conditions

The *A. thaliana* ecotype Columbia (Col-0) was used as the wild type. Seeds were sterilized and placed on 1/2 Murashige and Skoog medium in a growth chamber for germination; 7-day-old seedlings were transferred to soil and grown at 22°C, 65–80% humidity under long-day conditions (16 h/8 h light/dark). Three-week-old *A. thaliana* leaves were collected for bimolecular fluorescence complementation (BiFC) assay.

### Cloning Procedure

The complementary DNAs (cDNAs) of *Arabidopsis* CNGC11 (AT2G46440), CNGC12 (AT2G46450), CaM1 (AT5G37780), CaM2 (AT2G41110), CaM6 (AT5G21274), CaM7 (AT3G43810), CML8 (AT4G14640), CML9 (AT3G51920), CML10 (AT2G41090), and CML11 (AT3G22930) encoding full-length proteins were obtained by amplifying from the wild-type cDNA with gene-specific primers. For TEVC analysis in *Xenopus* oocytes, the PCR-amplified DNA fragments and the full open reading frames of *CaM1_12_*, *CaM1_34_*, and *CaM1_1234_* were inserted into the pGEMHE vector. For the subcellular localization assay, the full open reading frames of *CNGC12* and *CNGC11* were inserted into pCAMBIA 1302 to produce the 35S::CNGC11-GFP (or 35S::CNGC12-GFP) constructs. For BiFC assays in *Arabidopsis* mesophyll protoplasts, the full open reading frames of *CNGC12*, *CNGC11*, and *CaM1* were subcloned into the pSAT1-nVenus-N or pSAT1-cCFP-N vector to produce *CaMV35S::CNGC12-nVenus*, *CaMV35S::CNGC11-cCFP*, and *CaMV35S::CaM1-cCFP* with specific primers (Lee et al., 2008). For yeast two-hybrid (Y2H) experiments, each fragment of the *CNGC12* was subcloned into the pGBKT7 vector; the full open reading frames of *CaM1*, *CaM2*, *CaM6*, *CaM7*, *CML8*, *CML9*, *CML10*, and *CML11* were respectively inserted into the pGADT7 vector. For pull-down assays, the *GST-CaM1* construct was produced by inserting the full length of *CaM1* into the pGEX4T-1 vector. The cDNA encoding *CNGC12-CT* (441D-650*) was inserted into the pET28a vector to produce *His-CNGC12-CT*. The primers used in these cloning procedures are listed in **Table S1**.

### Y2H Assays

Pairs of each combination of pGBKT7 and pGADT7 vectors were co-transformed into yeast strain AH109 by the lithium acetate method (Ramer et al., 1992). The transformed cells were grown in liquid synthetic medium lacking tryptophan and leucine (−Trp, −Leu) and adjusted to an OD_600_ of 0.6. Cultures (8 µl) were spotted onto selection medium plates without tryptophan and leucine (−Trp, −Leu) and without histidine (−Trp, −Leu, −His). Selection plates were grown at 29°C for 2 days. Assays contained three replicates of each bait-and-prey combination.

### BiFC Assay and Subcellular Localization in *Arabidopsis* Protoplasts

For the BiFC assay, the plasmid combinations *nVenus-CNGC12*/*cCFP-CaM1*, *nVenus-CNGC12*/*cCFP*, *nVenus-CNGC12*/*cCFP-CNGC11*, and *nVenus*/*cCFP-CaM1* were transformed into protoplasts prepared from 3-week-old *A. thaliana* leaves (Abel and Theologis, 1994). The combination of *nVenus-CNGC12*/*cCFP* and *nVenus*/*cCFP-CaM1* was used as a negative control. For the subcellular localization assay, the expression constructs 35S::CNGC11-GFP, 35S::CNGC12-GFP, and 35S::GFP were transformed into *Arabidopsis* protoplasts. After incubation for 16–20 h at room temperature, the protoplasts were viewed, and images were captured using a Zeiss LSM510 confocal microscope and analyzed with LSM Image Browser software.

### Purification of Fusion Proteins

The full-length *CaM1* was constructed into the pGEX4T-1 vector, while the cDNA for *CNGC12* (*CT*) was subcloned into the pET28a vector to generate the pET28a*-CNGC12-CT* fusion construct. The vectors were expressed in the *Escherichia coli* strain Rosetta DE3 (Stratagene). When cells were grown to an OD_600_ of 0.6, they were induced with 1 mM isopropyl-β-d-thiogalactoside (IPTG) for 5 h at 37°C. For purification of the glutathione S-transferase (GST)-tagged fusion proteins, the cells were lysed by sonication on ice in phosphate-buffered saline (PBS) buffer containing protease inhibitor cocktail (Roche), 1 mM lysozyme, and 1% Triton X-100. The pellets and supernatants were separately collected by centrifugation at 12,000 *g* for 20 min at 4°C. The pellets were washed with the PBS solution four times. For GST fusion protein purification, the supernatants were purified using glutathione agarose beads (GE) and subjected to western blot with anti-GST antibody.

### GST Pull-Down Assays

Purified GST-CaM1 and GST proteins were individually incubated with glutathione agarose beads for 2 h at 4°C. The beads were collected after centrifugation and mixed with 1 ml of cell lysates containing His-tagged CNGC12-CT protein and then incubated at 4°C overnight. Centrifugation was performed after co-incubation, and the beads were collected and washed five times with PBS buffer. Finally, the GST bead-bound proteins were resuspended in a sodium dodecyl sulfate polyacrylamide gel electrophoresis (SDS-PAGE) sample buffer, boiled in 100°C water, and then immunoblot was performed with anti-His antibody (Abcam). His-CNGC12-CT protein was used as a control.

### Electrophysiological Procedures

Recording pipettes were pulled from borosilicate glass capillaries (Sutter Instruments, USA) by using a Flaming/Brown micropipette puller (model P-97, Sutter Instruments, USA). The capillary dimensions were 0.58 mm. The procedure is heat = 580, pull = 95, vel = 35, time = 90. The pipette solution included 3 M KCl. The capped RNA (cRNA) was transcribed by using the mMessage mMachine transcription kit (Ambion, Austin, TX). The quality of the cRNA was detected by denaturing gel electrophoresis. *Xenopus* oocytes were injected with 11.5 ng of cRNA, while an equivalent volume of water was injected as controls. Injected oocytes were incubated in ND96 solution at 18°C for 48 h, supplemented with 50 μg/ml gentamicin prior to electrophysiological assay. TEVC analysis was then performed using a TEV 200 amplifier (Dagan, Minneapolis, MN). Meanwhile, a Digidata 1440 A/D converter was used to monitor the whole process. The Clampex 10.2 software (Axon Instruments, Foster City, CA) was used for electrophysiological measurements. The bath solution contained 185 mM mannitol, 2 mM NaCl, 1 mM KCl, 2 mM MgCl_2_, 1.8 mM CaCl_2_, and 10 mM 2-(*N*-morpholino)ethanesulfonic acid (MES)-Tris (pH 5.5). Measurements of Ca^2+^ currents were performed under continuous perfusion solution containing 130 mM mannitol, 2.0 mM NaCl, 2.0 mM KCl, 30 mM CaCl_2_, and 5 mM MES-Tris (pH 5.5). For ionic selectivity analysis, the bath solutions respectively contained 30 mM Ca^2+^, 30 mM Mg^2+^, 30 mM Ba^2+^, 30 mM K^+^, and 30 mM Na^+^. The currents were recorded by membrane voltage steps potential at +40 to −140 mV (in 10-mV decrements, 4-s duration), and the holding potential was set to 0 mV.

## Results

### CNGC12 Is a Typical Ca^2+^-Permeable Channel and Mediates Inward Divalent Cationic Currents in *Xenopus* Oocytes

**Figure 1:**
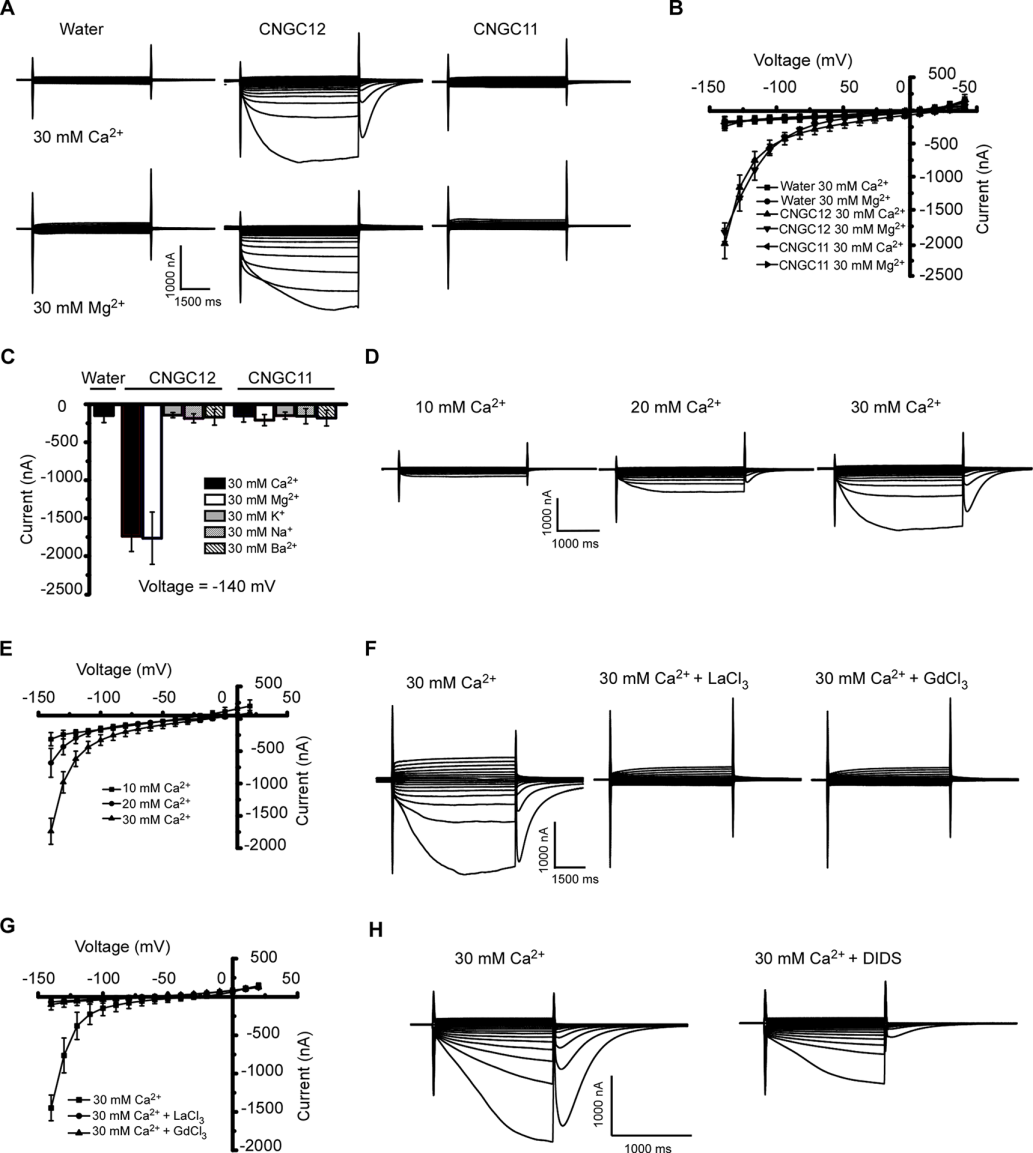
CNGC12 is a Ca^2+^-permeable, divalent cation-selective channel. **(A)** Typical whole-cell currents recorded from CNGC12- or CNGC11-expressing oocytes in bath solution containing 30 mM Ca^2+^ (upper set) or 30 mM Mg^2+^ (lower set) at pH 5.5; the water-injected oocytes were used as control. **(B)** Current–voltage (*I*–*V*) curves based on data from **(A)**. The data are expressed as means ± SE, with *n* = 5 for each group. **(C)** Ion selectivity of CNGC12 and CNGC11. The currents generated by CNGC12 and CNGC11 at −140 mV, when oocytes were perfused with a solution containing 30 mM Ca^2+^, 30 mM Mg^2+^, 30 mM Ba^2+^, 30 mM K^+^, or 30 mM Na^+^, pH = 5.5. The data are expressed as means ± SE, with *n* = 6 for each group. **(D)** Whole-cell currents recorded from CNGC12-expressing oocytes perfused with the bath solution containing different concentrations of CaCl_2_ (10, 20, or 30 mM). **(E)** Current–voltage (*I*–*V*) curves recorded as in **(D)**. The data are expressed as means ± SE, 10 mM, *n* = 4; 20 mM, *n* = 4; or 30 mM, *n* = 6. **(F)** Whole-cell currents recorded CNGC12-expressing oocytes when the oocytes are in a bath solution containing 30 mM Ca^2+^ and 100 μM LaCl_3_/100 μM GdCl_3_. **(G)** Current–voltage (*I*–*V*) curves recorded as in **(E)**. The data are expressed as means ± SE, with *n* = 5 for each group. **(H)** The inward currents were greatly inhibited by bath solution containing 30 mM Ca^2+^ and 100 μM 4,4′-diisothiocyanostilbene-2,2′-disulfonic acid (DIDS).

CNGC11 and CNGC12 have been reported to be involved in plant immune responses (Yoshioka et al., 2006; Urquhart et al., 2007; Chin et al., 2010; Urquhart et al., 2011; Moeder et al., 2019). However, their electrophysiological properties remain uncharacterized. To test whether they function as Ca^2+^-permeable channels that mediate Ca^2+^ influx across the PM, we expressed these proteins by injecting cRNA of CNGC12 or CNGC11 into *Xenopus* oocytes and followed by thorough whole-cell TEVC analysis. Compared with the water-injected control oocytes, the oocytes injected with CNGC12 cRNA showed an inward current in the presence of 30 mM extracellular Ca^2+^ or Mg^2+^, which indicated that the CNGC12 is active (**Figures 1A, B**). By contrast, no inward current was recorded when the oocytes injected with CNGC11 cRNA were placed in a solution containing 30 mM Ca^2+^ or 30 mM Mg^2+^ (**Figures 1A, B**). Further ionic selectivity analysis revealed that CNGC12 exhibited no detectable permeability to K^+^, Na^+^, and Ba^2+^ (**Figure 1C**). The oocytes expressing CNGC11 produced negligible currents in the bath solution containing 30 mM KCl, 30 mM NaCl, or 30 mM BaCl_2_ buffer, as did water-injected cells (**Figure 1C**). Tests in various Ca^2+^ concentrations (10, 20, and 30 mM) showed that the CNGC12 channel current was proportional to Ca^2+^ concentrations (**Figures 1D, E**). The CNGC12-mediated inward current was strongly decreased after application of 100 μM La^3+^ or 100 μM Gd^3+^ (**Figures 1F, G**). Our studies using CNGC11-GFP and CNGC12-GFP fusions showed that CNGC11 and CNGC12 proteins can be expressed and they were localized at the PM in *Xenopus* oocytes (**Figure S1**), which is consistent with the previously published data *in planta* (Baxter et al., 2008). Since it has been shown that *Xenopus* oocytes have a calcium-activated chloride channel (CaCC), which can be activated by increased intercellular Ca^2+^ (Hansen and Bräuner, 2009), both CNGC-mediated calcium influx and CaCC-mediated Cl efflux could contribute to the inward current. In agreement with this speculation, the inward currents were strongly reduced upon addition of 100 μM chloride channel blocker 4,4′-diisothiocyanostilbene-2,2′-disulfonic acid (DIDS) (**Figure 1H**). Together, these results indicate that CNGC12, but not CNGC11, functions as a Ca^2+^-permeable channel.

### The Activities of CNGC11 and CNGC12 Are Not Affected by cNMPs

**Figure 2:**
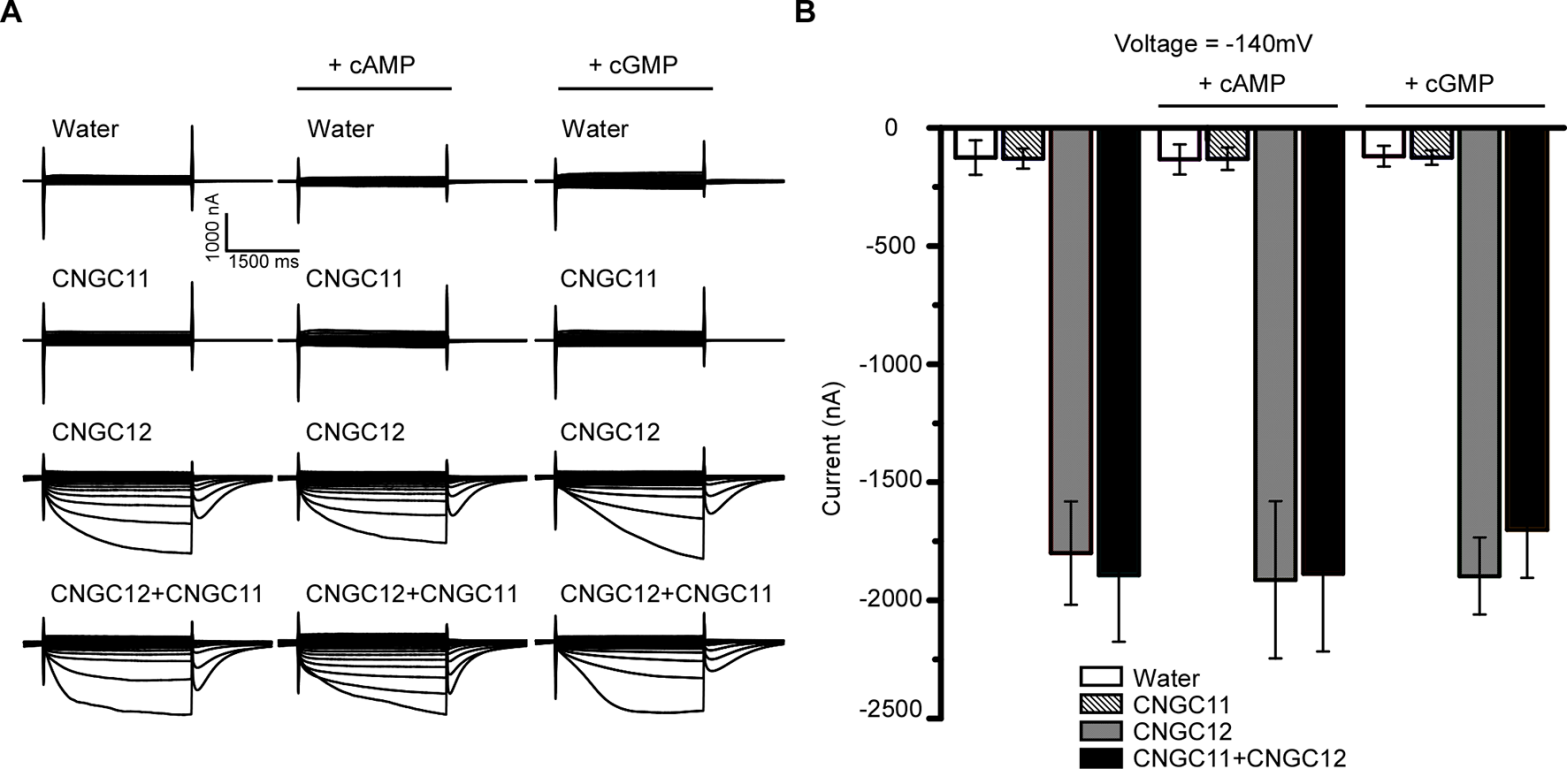
Cyclic nucleotide monophosphates (cNMPs) do not affect the activities of CNGC11 and CNGC12 in the *Xenopus* oocytes. **(A)** Typical current traces recorded in oocytes expressing water (control), CNGC12, CNGC11, CNGC12 plus CNGC11 (CNGC12 + CNGC11). The oocytes were perfused with a bath solution containing 30 mM Ca^2+^ (left), a bath solution containing 30 mM Ca^2+^ and 0.1 mM dibutyryl-cyclic AMP (cAMP) (middle), or the membrane-permeable cGMP analog 8-bromoguanosine 3′,5′-cyclic monophosphate (8Br-cGMP) (right). **(B)** Quantitative analysis of currents at −140 mV as in **(A)**. Values are expressed as mean ± SE, with *n* = 6 for each group.

Animal CNGCs are non-selective cation channels which are gated by the second messenger cNMPs (Brüggemann et al., 1993; Kaupp and Seifert, 2002), and some *Arabidopsis* CNGCs have been reported to function as a cyclic nucleotide-gated Ca^2+^-permeable channel (Leng et al., 2002; Ali et al., 2007; Gao et al., 2012; Wang et al., 2013). However, it is not clear whether other *Arabidopsis* CNGCs are also gated by cNMPs. To test the effect of cNMPs on the channel activities of CNGC11 and CNGC12, we expressed CNGC11 and CNGC12 in *Xenopus* oocytes and measured their activities by TEVC recording. As shown earlier, CNGC12 alone was active, and CNGC11 alone was inactive. Similar current amplitudes were observed upon extracellular application of 0.1 mM dibutyryl-cAMP or the membrane-permeable cGMP analog 8-bromoguanosine 3′,5′-cyclic monophosphate (8Br-cGMP) (**Figures 2A, B**), suggesting that cAMP or cGMP did not affect the activities of CNGC12 or CNGC11. Since CNGC8 and CNGC18 can form a heterotetramer channel (Pan et al., 2019), we asked whether CNGC11 and CNGC12 can form a heteromeric channel too. To test this hypothesis, we co-expressed CNGC11 and CNGC12 in oocytes and recorded the current in a bath solution containing 30 mM Ca^2+^. We found that the oocytes co-expressing CNGC11 and CNGC12 produced inward currents similar to those of oocytes expressing CNGC12 alone (**Figures 2A, B**). Moreover, upon extracellular application of 0.1 mM dibutyryl-cAMP or 8Br-cGMP, the current amplitudes in CNGC11- and CNGC12-co-expressing oocytes were not affected (**Figures 2A, B**). Taken together, our data suggest that, different from their animal counterparts, plant CNGCs may not be gated by cNMPs.

### CNGC12 Physically Interacts With CaM1

**Figure 3:**
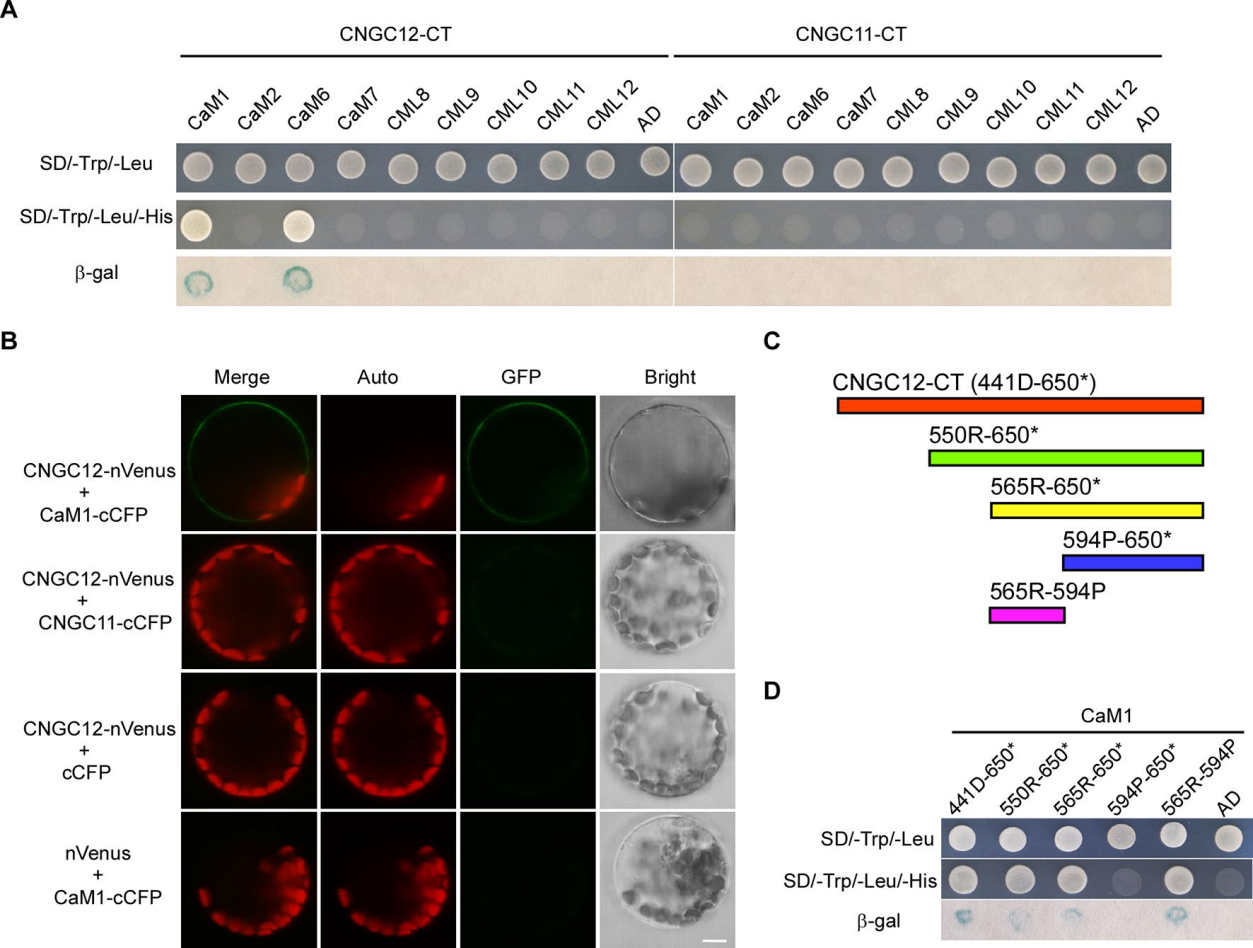
CaM1 physically interacts with CNGC12. **(A)** Yeast two-hybrid assay of calmodulins (CaMs)/CaM-like proteins (CMLs) and the C-terminal of CNGC12 (CNGC12-CT) and CNGC11 (CNGC11-CT). The combination of pGBKT7-CNGC12-CT/pGBKT7-CNGC11-CT and empty pGADT7 vectors were used as a negative control. SD/−Trp/−Leu represents synthetic dextrose minimal medium without tryptophan and leucine; SD/−Trp/−Leu/−His indicates synthetic dextrose minimal medium without tryptophan, leucine, and histidine. Growth of yeast on SD/−Trp/−Leu/−His plate indicates interaction between the two tested proteins. **(B)** Bimolecular fluorescence complementation (BiFC) analysis between CNGC12 and CaM1 or CNGC11 in *Arabidopsis* mesophyll protoplasts. Vectors encoding CNGC12-nVenus, CaM1-cCFP, CNGC11-cCFP, and nVenus were co-expressed in various combinations in *Arabidopsis* protoplasts. Scale bars, 5 μm. **(C)** Diagram representing various domains of the C-terminus of CNGC12 (CNGC12-CT) which were cloned and used as prey during yeast two-hybrid screening: 441D-650* (the C-terminal of CNGC12); 550R-650* [the C-terminal of CNGC12 without the first cyclic nucleotide-binding domain (CNBD)]; 565R-650* (the C-terminal of CNGC12 without two CNBDs); 565R-594P (IQ domain); 594P-650* (after the IQ domain to the end of CNGC12). **(D)** Yeast two-hybrid analysis of CaM1 with various fragments of CNGC12-CT.

One typical feature of plant CNGC proteins are their CaM-binding sites. It has been reported that CNGC12 can bind CaMs *via* multiple CaMBDs at both N- and C-terminal cytosolic regions and is regulated positively and negatively by CaMs (DeFalco et al., 2016). However, CaM binding has not been studied well with CNGC11, and also, it is not clear whether specific CaMs are binding to these CNGCs. Thus, we have conducted Y2H analysis with six different *Arabidopsis* CaMs and CMLs. First, Y2H analysis revealed that CNGC12 interacted with CaM1 and CaM6 (**Figure 3A**). Since CaM6 showed no regulation on the activity of CNGC12, while CaM1 did (see below), our study further focused on CaM1. Next, *in vitro* pull-down assays were carried out by incubating GST-tagged CaM1 and the His-tagged cytosolic domain of CNGC12 (CNGC12-CT). SDS-PAGE and western blots were used to analyze the co-purified proteins. Results showed that the C-terminal cytosolic domain of CNGC12 binds the purified CaM1-GST protein, but not the GST peptide alone (**Figure S2**). Then, BiFC assays were carried out in *Arabidopsis* protoplasts. The combination of CNGC12-nVenus and CaM1-cCFP produced a green fluorescent signal in the PM of *Arabidopsis* protoplasts, indicating that CNGC12 can interact with CaM1 in the PM of plant cells (**Figure 3B**). CNGC12 showed no interaction with CNGC11 (**Figure 3B**). Together, these results suggest that CNGC12 can physically interact in plant cells with CaM1. The interaction between CNGC12 and CaM1 is similar to the earlier report (Fischer et al., 2017).

To map the CaM1 interaction domain of CNGC12, the binding abilities of different truncated C-terminal fragments [**Figure 3C**; 441D-650* (the C-terminus of CNGC12), 550R-650* (the C-terminus of CNGC12 without the first CNBD), 565R-650* (the C-terminus of CNGC12 without two CNBDs), 565R-594P (IQ domain), 594P-650* (after the IQ domain to the end of CNGC12)] were analyzed in the Y2H system. The peptide fragments that include the amino acids corresponding to the IQ domain (565R-594P) were able to interact with CaM1 (**Figure 3D**). These results are further evidence of the physical interaction between CaM1 and CNGC12 and show that the interacting region is the IQ domain.

### The Activity of CNGC12 Is Enhanced by CaM1

**Figure 4:**
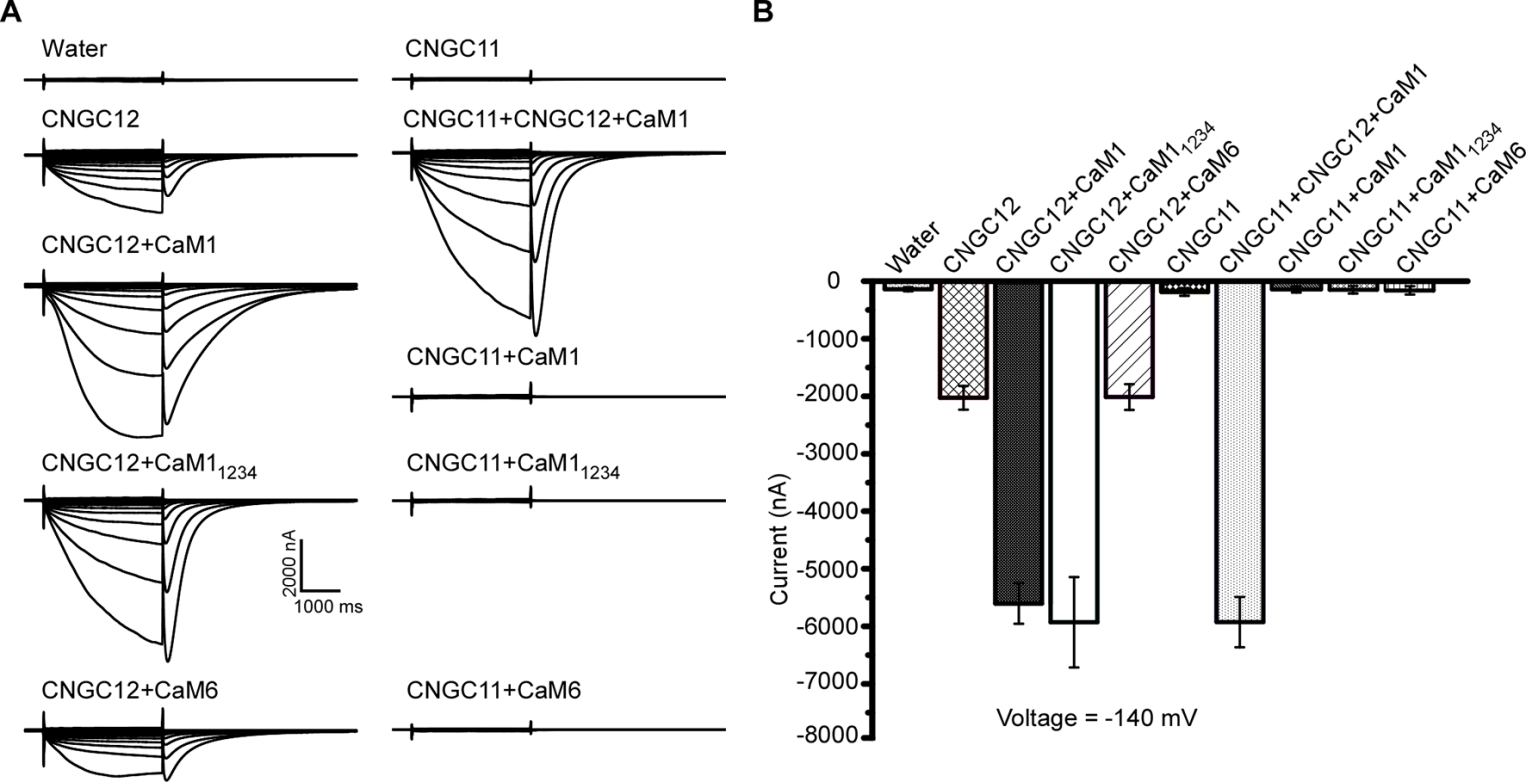
CaM1 activates CNGC12 in *Xenopus* oocytes. **(A)** Whole-cell currents were recorded from the oocytes injected with water (control), CNGC12, CNGC12 plus CaM1 (CNGC12 + CaM1), CNGC12 plus CaM1_1234_ (CNGC12 + CaM1_1234_), CNGC12 plus CaM6 (CNGC12 + CaM6), CNGC11, CNGC1 plus CNGC12 and CaM1 (CNGC11 + CNGC12 + CaM1), CNGC11 plus CaM1 (CNGC11 + CaM1), CNGC11 plus CaM1_1234_ (CNGC11 + CaM1_1234_), and CNGC11 and CaM6 (CNGC11 + CaM6). The oocytes were perfused with bath solution containing 30 mM Ca^2+^ (left). **(B)** Effects of CaM1, CaM1_1234_, and CNGC11 on the currents generated by CNGC12 at −140 mV. The pooled current values were at 1.8 s of each voltage-clamp episode. The data are expressed as means ± SE. (water, *n* = 6; CNGC12, *n* = 6; CNGC12 + CaM1, *n* = 8; CNGC12 + CaM1_1234_, *n* = 5; CNGC12 + CaM6, *n* = 7; CNGC11, *n* = 6; CNGC11 + CNGC12 + CaM1, *n* = 6; CNGC11 + CaM1, *n* = 6; CNGC11 + CaM1_1234_, *n* = 6; and CNGC11 + CaM6, *n* = 8).

Previous studies have shown that CaM binding at the IQ domain positively regulates CNGC12 (DeFalco et al., 2016), and in our earlier assays, CaM1 and CaM6 physically interacted with CNGC12; thus, we subsequently tested if the activity of CNGC12 is regulated by CaM1 or CaM6. Firstly, when CaM1 and CNGC12-GFP fusions were co-expressed in oocytes, we found that co-expression of CaM1 with CNGC12-GFP did not affect the expression level of CNGC12-GFP fusion at the PM. Then, we measured the channel activity in CNGC12- and CaM1-co-expressing *Xenopus* oocytes. While CNGC12 expression alone in the oocytes produced a detectable current under continuous perfusion with a bath solution containing 30 mM extracellular Ca^2+^ (**Figures 4A, B**), the channel activity was significantly enhanced when CNGC12 was co-expressed with CaM1 (**Figures 4A, B**). As a prototypical EF hand protein, CaM contains four Ca^2+^-binding EF hand motifs (Babu et al., 1985). When the four EF hand motifs were mutated by replacing the key residue (E) for Ca^2+^ binding in each EF hand, the mutant CaM (called CaM_1234_) became incapable of binding Ca^2+^ (Haiech et al., 1991). We generated the mutant form of CaM1, CaM1_1234_, with all four EF hands mutated. Interestingly, when co-expressed with CaM1_1234_, the CNGC12 channel activity was also dramatically enhanced (**Figures 4A, B**). These results indicate that CaM1 enhances the channel activity of CNGC12 in a Ca^2+^-independent way. The current amplitude in CNGC12- and CaM6-co-expressing oocytes is similar to that in oocytes expressing CNGC12 alone, suggesting that CaM6 does not affect the channel activity of CNGC12 (**Figures 4A, B**). We co-expressed CNGC11, CNGC12, and CaM1 in oocytes and found that the currents were similar to the oocytes co-expressing CNGC12 and CaM1 (**Figures 4A, B**). CNGC11 showed negligible inward currents when co-expressed with CaM1, CaM1_1234_, or CaM6 (**Figures 4A, B**). These electrophysiological data demonstrated that CaM1 specifically enhanced the activity of CNGC12, possibly in a Ca^2+^-independent manner, which is in line with the previous findings that CaM binding to CNGC12 is required for the function of the channel (DeFalco et al., 2016).

## Discussion

Previous studies have shown that CNGC11 and CNGC12 participated in Ca^2+^ transport using a yeast heterologous expression system (Urquhart et al., 2007; Chin et al., 2010). In this study, we expressed CNGC11 and CNGC12 in oocytes and tested whether both proteins could mediate divalent cationic currents across the PM by TEVC technology. Our results in this paper show that CNGC12 is an active calcium channel electrophysiologically in *Xenopus* oocytes (**Figures 1A–H**). The ionic selectivity analysis showed that CNGC12 preferentially transports divalent cations such as Ca^2+^ and Mg^2+^ (**Figures 1A–C**); the CNGC12 channel current is proportional to Ca^2+^ concentrations (**Figures 1D, E**); and the activity of CNGC12 is inhibited by typical calcium channel inhibitors (**Figures 1F, G**). These electrophysiological properties suggest that CNGC12 is a typical Ca^2+^-permeable channel. Different from the previous results showing that both CNGC11 and CNGC12 work as Ca^2+^ channels using yeast functional complementation analysis (Urquhart et al., 2007; Chin et al., 2010; Urquhart et al., 2011), our electrophysiological study using *Xenopus* oocytes indicates that CNGC11 does not transport Ca^2+^, Mg^2+^, Ba^2+^, K^+^, or Na^+^ (**Figure 1C**). It indicates that CNGC11 is inactive or the activation mechanism is different from CNGC12 in oocytes. It may be that the channel activity or regulation of CNGC11 requires other subunits. The function of CNGC11 needs to be further investigated in the future.

The animal CNGCs are allosterically regulated when the cNMPs cAMP and cGMP interact with the C-terminal CNBD (Brüggemann et al., 1993; Kaupp and Seifert, 2002). In this study, we found that cAMP does not affect the activities of CNGC11 or CNGC12 in oocytes (**Figures 2A, B**). Consistent with the earlier report (Yoshioka et al., 2006), the activity of CNGC11 or CNGC12 is not activated by cGMP either (**Figures 2A, B**). In addition, when CNGC11 and CNGC12 were co-expressed in oocytes, cAMP or cGMP did not affect their channel activities (**Figures 2A, B**). These findings suggest that plant CNGCs may not be gated by cNMPs, which are different from their animal counterparts. Previous studies have shown that CNGC8 and CNGC18 can form a heterotetramer channel (Pan et al., 2019); we tested whether CNGC11 and CNGC12 can form a heteromeric channel as well. Our study here shows that CNGC11 does not affect the activity of CNGC12 (**Figures 2A, B**) and CNGC11 also does not interact with CNGC12 in *Arabidopsis* protoplasts (**Figure 3B**). Transient expression of 35S::CNGC11-GFP and 35S::CNGC12-GFP fusion constructs in *Arabidopsis* protoplasts revealed that CNGC11 and CNGC12 are targeted to the PM, respectively (**Figure S3**), which is consistent with the earlier report (Baxter et al., 2008). These results indicate that CNGC11 and CNGC12 may not form a heteromeric channel in oocytes.

A large variety of Ca^2+^-permeable channels are known to be regulated by the Ca^2+^-binding protein CaM. CNGC12 has multiple CaMBDs at its N- and C-termini, which is similar to animal CNGC isoforms (Arazi et al., 2000; Ungerer et al., 2011) but unlike any plant channel studied to date (Köhler and Neuhaus, 2000; Hua et al., 2003; DeFalco et al., 2016). While the IQ domain and N- and C-termini of CNGC12 can interact with CaM and these CaM binding sites can both positively and negatively regulate CNGC12 (DeFalco et al., 2016), the current model on CaM regulation of plant CNGCs is difficult to prove. Our results in this paper show that CaM1 interacts with CNGC12 (**Figures 3A, B** and **S2**) and the interacting region is the IQ domain (**Figure 3C**), which is similar as reported earlier (DeFalco et al., 2016). In this study, our electrophysiological studies show that the channel activity of CNGC12 is dramatically enhanced by co-expression with CaM1 (**Figures 4A, B**), which help to elucidate the complex mode of CaM1-mediated regulation of CNGC12. Although the amino acid sequences of *Arabidopsis* CaMs are very similar (McCormack et al., 2005), the CaMs exhibit various expression patterns, and they are reported to participate in different physiological processes *via* regulating different CNGCs (Pan et al., 2019; Tian et al., 2019), and our study also shows that only CaM1 enhanced the activity of CNGC12. These findings suggest that the regulation of CNGC channels by CaMs may be specific. Further studies should be carried out to examine if CaM1 regulates CNGC12 *in vivo*. Considering the functional diversity of CNGCs in plant development and stress response, it will be interesting to test if CNGC11 or CNGC12 also functions in some other physiologic processes besides plant immunity and heavy metal ion uptake.

## Author Contributions

LL and HZ conceived the project. ZZ, WT and CH designed and performed the research. ZZ and CH analyzed the data and wrote the paper. LL, WT and HZ revised the paper.

## Funding

This work was supported by the National Key Research and Development and Program of China (Grant YFD0300102-3 to LL), National Science Foundation of China (31470365 and 21370927 to LL; 31570265 to ZH).

## Conflict of Interest Statement

The authors declare that the research was conducted in the absence of any commercial or financial relationships that could be construed as a potential conflict of interest.

## Supplementary Material

Figure S1 CNGC11-GFP, CNGC12-GFP, and CaM1 + CNGC12-GFP are expressed in oocytes and localized at the plasma membrane. The *Xenopus* oocytes were injected with the capped RNA (cRNA) of CNGC11-GFP, CNGC12-GFP, and CaM1 + CNGC12-GFP. Each group shows a quarter of an oocyte.

Figure S2 CaM1 physically interacts with CNGC12. Glutathione S-transferase (GST) pull-down assay. GST pull-down analysis of the interaction between CNGC12 and CaM1; GST-apo pull-down was used as control.

Figure S3 CNGC11 and CNGC12 are localized at the plasma membrane. 35S::CNGC11-GFP, 35S::CNGC11-GFP, and 35S::GFP were transiently expressed in *Arabidopsis* mesophyll protoplasts. GFP was used as a control. Bar = 5 μm.
