# Supplementary figures and images for: Electrophysiological Studies Revealed CaM1-Mediated Regulation of the Arabidopsis Calcium Channel CNGC12
Source: Front Plant Sci. 2019 Sep 10;10:1090. doi: 10.3389/fpls.2019.01090 (PMC6749817; doi:10.3389/fpls.2019.01090)

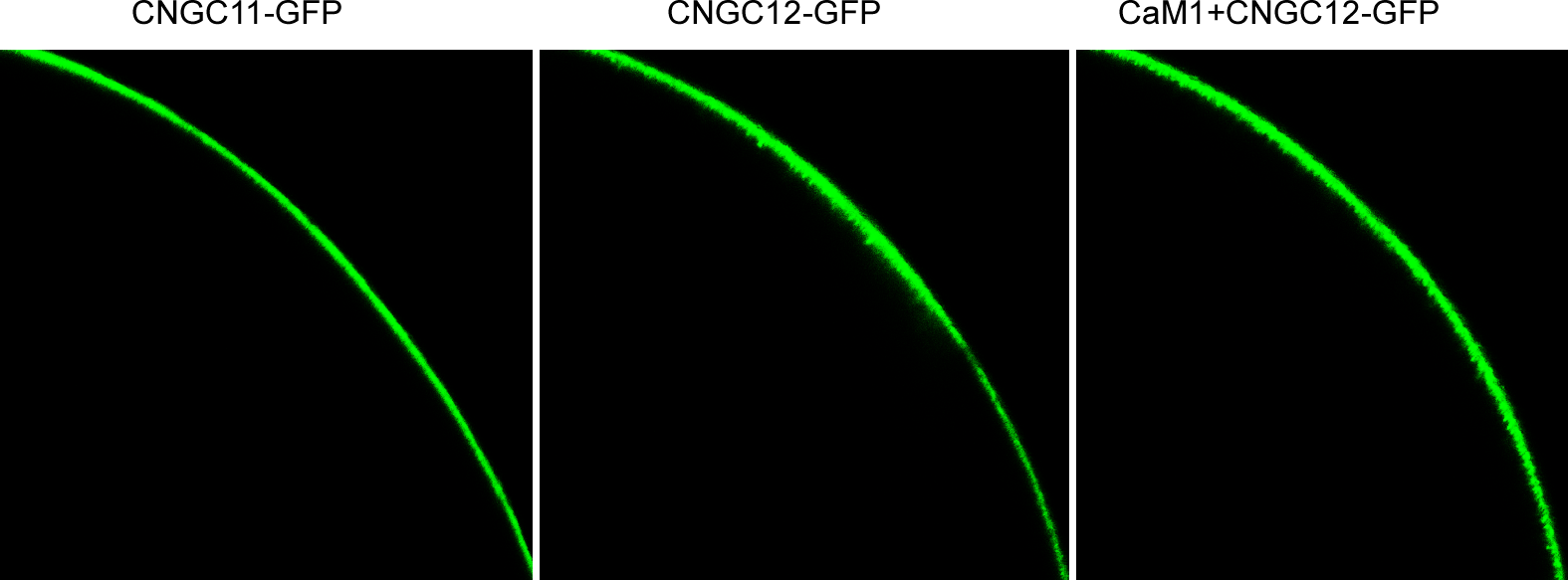

Supplement: Figure S1 — CNGC11-GFP, CNGC12-GFP, and CaM1 + CNGC12-GFP are expressed in oocytes and localized at the plasma membrane. The Xenopus oocytes were injected with the capped RNA (cRNA) of CNGC11-GFP, CNGC12-GFP, and CaM1 + CNGC12-GFP. Each group shows a quarter of an oocyte. [file Image_1.tif]

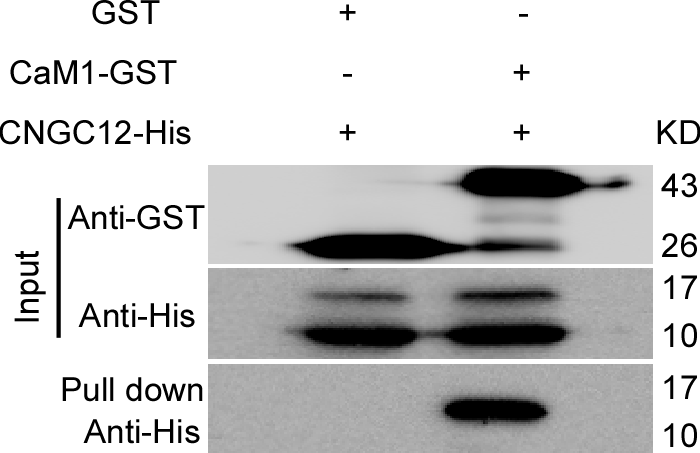

Supplement: Figure S2 — CaM1 physically interacts with CNGC12. Glutathione S-transferase (GST) pull-down assay. GST pull-down analysis of the interaction between CNGC12 and CaM1; GST-apo pull-down was used as control. [file Image_2.tif]

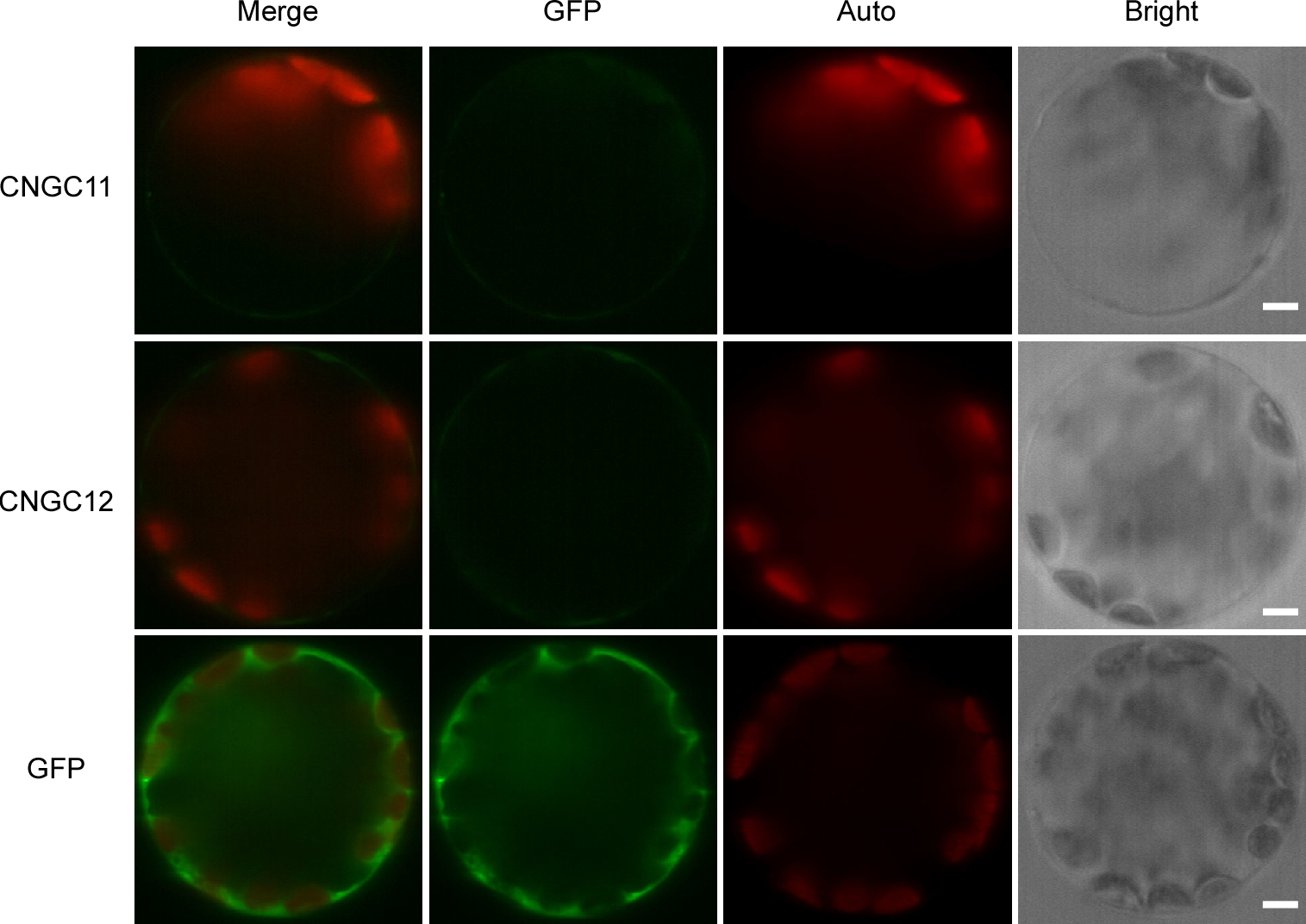

Supplement: Figure S3 — CNGC11 and CNGC12 are localized at the plasma membrane. 35S::CNGC11-GFP, 35S::CNGC11-GFP, and 35S::GFP were transiently expressed in Arabidopsis mesophyll protoplasts. GFP was used as a control. Bar = 5 μm. [file Image_3.tif]
