# Supplementary material for: Electrophysiological Studies Revealed CaM1-Mediated Regulation of the Arabidopsis Calcium Channel CNGC12
Source: Front Plant Sci. 2019 Sep 10;10:1090. doi: 10.3389/fpls.2019.01090 (PMC6749817; doi:10.3389/fpls.2019.01090)
Supplement: Supplementary file 4 [file Table_1.docx]

**Table S1. Primers used for cloning**

| **Gene name** | **Forward primer 5’-3’** | **Reverse primer 5’-3’** | **Enzyme sites** |
| --- | --- | --- | --- |
| CNGC11 | ATGAATCTTCAGAGGAGAAAAT | CTACGCATAAATCGCAGCACCT | XmaI/XbaI for pGEMHE  KpnI/BamHI for pSAT1-cCFP-N  NcoI/SpeI for pCAMBIA1302 |
| CNGC11(CT) | CAATCATCTACG ACTAGAGTAG | CTACGCATAAATCGCAGCACCTA | ECORI/BamHI for pGBDT7 |
| CNGC12 | ATGAATCATCGGAGGAGTA | CTATGCTTCAGCCTTTGCA | BamHI/XbaI for pGEMHE  EcoRI/XmaI for pSAT1-nVenus-N  BglII/SpeI for pCAMBIA1302 |
| CNGC12(CT) | CAATCATCTACTACTAGAGT | CTATGCTTCAGCCTTTGCA | BamHI/EagI for pET28a |
| CNGC12(441) | GATGGTTGGCTACTAGAAGCT | CTATGCTTCAGCCTTTGCA | ECORI/BamHI for pGBDT7 |
| CNGC12(550) | AGACAAAAACTCCAACGAACAT | CTATGCTTCAGCCTTTGCA | ECORI/BamHI for pGBDT7 |
| CNGC12(565) | CGCTCATGGGCAGCATTCTTC | CTATGCTTCAGCCTTTGCA | ECORI/BamHI for pGBDT7 |
| CNGC12(594) | CCACAAGGCACGCAACTCAAT | CTATGCTTCAGCCTTTGCA | ECORI/BamHI for pGBDT7 |
| CNGC12  (565-594) | AGACAAAAACTCCAACGAACAT | ATTGAGTTGCGTGCCTTGTGG | ECORI/BamHI for pGBDT7 |
| CaM1 | ATGGCGGATCAACTCACTGAC | TCACTTAGCCATCATAATCTT | NdeI/BamHI for pGADT7  BamHI/XbaI for pGEMHE  SmaI/ NotI for pGEX-4T-1  KpnI/BamHI for pSAT1-cCFP-N |
| CaM1_1_(D21A) | TAGCCTCTTCGCCAAAGATGGCGA | TCGCCATCTTTGGCGAAGAGGCTA | pGADT7 (For point mutation) |
| CaM1_2_(D57A) | TCAACGAGGTTGCTGCAGATGG | CCATCTGCAGCAACCTCGTTGA | pGADT7 (For point mutation) |
| CaM1_3_(D94A) | AGGGTTTTCGCCAAAGACCAG | CTGGTCTTTGGCGAAAACCCT | pGADT7 (For point mutation) |
| CaM1_4_(D130A) | CCGTGAGGCTGCTGTTGATGGA | TCCATCAACAGCAGCCTCACGG | pGADT7 (For point mutation) |
| CAM2 | ATGGCGGATCAGCTCACAGACGA | TCACTTAGCCATCATAACCTTC | Nco I/ BamH I for pGADT7 |
| CAM6 | ATGGCGGATCAGCTCACCGATGACCAGATCTCAG | TCACTTAGCCATCATGACTTTGACGAATTCTTCATAG | XmaI/BamHI for pGADT7  BamHI/XbaI for pGEMHE |
| CAM7 | ATGGCGGATCAGCTAACCGATGACCAGATCTCCGAG | TCACTTTGCCATCATGACTTTGACGAACTCTTCATAG | EcoRI/BamHI for pGADT7 |
| CML8 | ATGGAAGAAACAGCACTGACAAAAGAT | TCAGTCAATGTTGATCATCATCTTGAC | EcoRI/BamHI for pGADT7 |
| CML9 | ATGGCGGATGCTTTCACAGATGAACAG | CTAATAAGAGGCAGCAATCATCATTTTAG | XmaI/BamHI for pGADT7 |
| CML10 | ATGGCGAATAAGTTCACTAGACAAC | TCAAGAAAACAACGCTTCGAACAAAT | EcoRI/BamHI for pGADT7 |
